# Circulating IGFBP-7 in Obstructive Sleep Apnea: A Single-Cohort Preliminary Assessment of Its Diagnostic Performance and Post-Treatment Dynamics

**DOI:** 10.3390/biomedicines14081780

**Published:** 2026-08-07

**Authors:** Abdulmohsen Alterki, Eman AlShawaf, Mohammad Alawadh, Dalal Shamiyah, Irina Alkhairi, Preethi Cherian, Devarajan Sriraman, Mahmoud Ebrahim, Mohammed Alterki, Saadoun Bin-Hasan, Fahd Al-Mulla, Mohamed Abu-Farha, Jehad Abubaker

**Affiliations:** 1 Department Otolaryngology, Head & Neck Surgery Faculty, Zain & Al Sabah Hospitals, Kuwait City 13083, Kuwait; abdulmohsen.alterki@dasmaninstitute.org; 2 Kuwait Board of Post Graduate Training Program, Department Otolaryngology, Head & Neck Surgery Faculty, Kuwait Institute for Medical Specializations (KIMS), Kuwait City 12050, Kuwait; 3 Research Sector, Dasman Diabetes Institute, Kuwait City 15462, Kuwait; 4 Department of Biochemistry and Molecular Biology, Dasman Diabetes Institute, Kuwait City 15462, Kuwait; eman.alshawaf@dasmaninstitute.org (E.A.); mohammadalawadh22@rcsi.ie (M.A.); dalalshamiyah@gmail.com (D.S.); irina.alkhairi@dasmaninstitute.org (I.A.); preethi.cherian@dasmaninstitute.org (P.C.); 5 Special Service Facility Department, Dasman Diabetes Institute, Kuwait City 15462, Kuwait; sriraman.devarajan@dasmaninstitute.org; 6 Department of Otolaryngology, McGill University, Montreal, QC H3A 0G4, Canada; ma7moud_ibrahim@hotmail.com; 7 Department of General Surgery, Farwaniya Hospital, Ministry of Health, Kuwait City 13110, Kuwait; alterkimohammed@gmail.com; 8 Sleep Medicine and Pediatric Respirology, Farwaniya Hospital, Kuwait City 13110, Kuwait; saadoun.binhasan@icloud.com; 9 Translational Research Department, Dasman Diabetes Institute, Kuwait City 15462, Kuwait; fahd.almulla@dasmaninstitute.org

**Keywords:** obstructive sleep apnea, hypoxia, IGFBP-7, IGFBP4, multilevel surgery, obesity, biomarker

## Abstract

**Background/Objectives**: Obstructive sleep apnea (OSA) is a sleep condition characterized by intermittent hypoxia, systemic inflammation, and metabolic dysfunction. Contemporary diagnosis criteria depend on polysomnography (PSG), a procedure that is limited by cost and accessibility. Identifying reliable circulating biomarkers may facilitate disease diagnosis and treatment monitoring. **Methods**: Insulin-like growth factor binding protein-7 (IGFBP-7) has been implicated in pathways relevant to OSA, although its precise role remains elusive. While we previously identified IGFBP-7 as a candidate of interest, in this report, we assessed circulating IGFBP-7 levels in a single-cohort study of 164 participants (124 with OSA, 40 controls) at the Dasman Diabetes Institute. A Type I PSG test was performed in a level 1 sleep laboratory to diagnose sleep apnea. Among the 124 patients with OSA who underwent multilevel sleep surgery (MLS), 67 completed the 3-month postoperative follow-up and were included in the longitudinal analyses. In these participants, we evaluated the apnea–hypopnea index (AHI) at baseline and 3 months after surgery and examined the associations between circulating IGFBP-7 levels, OSA severity indices, and metabolic parameters. **Results**: Our data revealed a significant increase in IGFBP-7 levels in people with OSA compared to controls (*p* < 0.001). Importantly, the increase in IGFBP-7 was positively correlated with AHI (r = 0.272, *p* < 0.001), indicating a potential link with this condition. IGFBP-7 levels declined significantly following MLS (*p* < 0.001), paralleling improvements in AHI and suggesting responsiveness to therapeutic intervention and a reduced hypoxic burden. IGFBP-4 levels increased significantly in patients with OSA (*p* = 0.006) but were not correlated with IGFBP-7. The receiver operating characteristic (ROC) analysis identified IGFBP-7 with a cut-off value of 14,003.21 pg/mL as a potential biomarker for OSA, with moderate performance (AUC = 0.722, 95% CI: 0.636–0.808; sensitivity 73%, specificity 84%). Notably, combining IGFBP-7 with IGFBP-4 resulted in a modest improvement (AUC = 0.755). **Conclusions**: Our data suggest that IGFBP-7 shows a modest association with OSA severity and may have exploratory value as part of a multi-marker or risk-stratification approach. Further large-scale and longitudinal studies are warranted to extensively explore the potential utility of IGFBP-7 within multi-marker approaches.

## 1. Introduction

Obstructive sleep apnea (OSA) is a prevalent and frequently underdiagnosed sleep-related breathing disorder characterized by recurrent partial or complete collapse of the upper airway during sleep [1]. Repeated episodes of airflow obstruction trigger systemic pathophysiological disturbances that extend beyond the respiratory system. A growing body of evidence links OSA to major systemic complications, including hypertension, coronary artery disease, stroke, heart failure, and metabolic disturbances, including insulin resistance and type 2 diabetes mellitus [1,2,3]. In addition to these cardiometabolic consequences, chronic sleep disruption contributes to neurocognitive impairment, excessive daytime sleepiness, and an increased risk of motor vehicle accidents, collectively representing a substantial public health and economic burden [2,4].

At the molecular level, the pathophysiology of OSA is largely driven by intermittent hypoxia (IH), characterized by repetitive cycles of oxygen desaturation and reoxygenation that resemble ischemia–reperfusion injury [5]. These fluctuations promote excessive generation of reactive oxygen species (ROS), resulting in oxidative stress and activation of hypoxia-responsive and inflammatory signalling pathways [5,6,7]. The resulting cellular stress alters the expression of numerous circulating proteins involved in metabolic regulation, inflammation, and vascular homeostasis [3,8].

The current clinical practice relies primarily on polysomnography (PSG) for OSA diagnosis. Although PSG remains the gold-standard method for assessing sleep architecture and calculating the apnea–hypopnea index (AHI) [8], it is a technically demanding and expensive procedure [9]. These limitations lead to substantial underdiagnosis, with a large proportion of OSA cases remaining unidentified and untreated [4]. Therefore, there is growing interest in identifying peripheral biomarkers that could serve as rapid, non-invasive, and cost-effective tools for screening, risk stratification, and monitoring treatment response [3,8].

Several peripheral biomarkers have been investigated as potential tools to complement conventional OSA diagnostics [8]. A recent systematic review identified several promising candidates for OSA diagnosis, including *ADAM29*, *FLRT2*, and *SLC18A3* mRNA levels in peripheral blood mononuclear cells; serum markers such as Endocan and YKL-40; and plasma biomarkers such as IL-6 and Vimentin [8]. Additionally, emerging evidence suggests that circulating microRNAs may serve as sensitive and non-invasive biomarkers for OSA, reflecting underlying changes in gene regulation and cellular stress responses [10]. Proteins involved in metabolic and hypoxia-related pathways have also been explored, with insulin-like growth factor binding protein-4 (IGFBP-4) and the glycoprotein follistatin-like 1 (FSTL1) demonstrating potential diagnostic value [11,12,13]. Collectively, these findings highlight the potential of molecules involved in metabolic regulation and cellular stress as biomarkers for OSA.

Among such molecules, insulin-like growth factor binding protein-7 (IGFBP-7) has emerged as a circulating biomarker of senescence, cellular stress and metabolic dysfunction [14]. IGFBP-7 is a secreted protein in the insulin-like growth factor binding protein superfamily and modulates growth factor and metabolic signalling pathways [15]. Clinically, it is best known as part of the FDA-approved TIMP-2/IGFBP-7 panel for early prediction of acute kidney injury in critically ill patients [16,17], and elevated levels have also been associated with adverse cardiovascular outcomes [18,19]. Given that OSA is characterized by intermittent hypoxia and systemic cellular stress [3,5], IGFBP-7 is a biologically plausible biomarker linked to these processes. Notably, IGFBP-4, a member of the same superfamily, has been shown to be significantly associated with OSA, further supporting the relevance of this protein family [11]. IGFBP-7 emerges as a compelling yet indirect mechanistic connection to OSA, due to its position in several OSA-induced pathogenic pathways. Studies have shown intermittent hypoxia to activate NF-kB signalling in endothelial cells, and IGFBP-7 was found to act as an active amplifier of this inflammatory response, exacerbating barrier dysfunction and promoting apoptosis [20,21,22]. Additionally, IGFBP-7 production was found to be directly stimulated by oxLDL [23], which is known to increase in OSA. This increase was found to inhibit the beneficial SIRT1 pathway, leading to endothelial apoptosis and inflammation [23]. This converging mechanistic evidence, encompassing NF-kB amplification, senescence propagation, and endothelial dysfunction, strongly implicates IGFBP-7 in the pathophysiology of OSA [24]. However, direct human studies measuring IGFBP-7 in patients with OSA and correlating it with disease severity or treatment response are lacking. This highlights IGFBP-7’s potential significance and testable gap in the literature.

Despite increasing interest in biomarker discovery for OSA, current data remains limited. Much of the available evidence depends on relatively small, single-centre studies that often lack standardized protocols [8]. Moreover, given the considerable phenotypic and endotypic heterogeneity of OSA, it is unlikely that any single biomarker will provide adequate diagnostic accuracy on its own [8,12]. Consequently, continued efforts to identify biologically relevant and clinically actionable biomarkers are required. Such markers may ultimately contribute to composite biomarker panels that improve diagnosis.

Because intermittent hypoxia underlies both inflammatory activation and neurophysiological dysfunction in OSA, we additionally evaluated the relationship between IGFBP-7 and two complementary hypoxia-related mediators. TNF-α was selected as a marker of hypoxia-induced systemic inflammation through NF-κB activation, whereas Orexin-A was included because of its established role in regulating wakefulness, respiratory drive, and upper airway muscle tone, all of which are disrupted in OSA [3].

The present study aimed to investigate circulating IGFBP-7 levels in patients with OSA. Specifically, we sought to compare IGFBP-7 levels between individuals with OSA and non-OSA controls, assess changes following MLS intervention, and examine potential associations between IGFBP-7 and selected metabolic and hypoxia-related mediators, including Orexin-A, TNF-α, and IGFBP4. Consequently, this will aid our understanding of the potential utility of IGFBP-7 as a diagnostic and treatment-monitoring biomarker in OSA.

## 2. Materials and Methods

### 2.1. Study Population and Sample Collection

**Figure 1:**
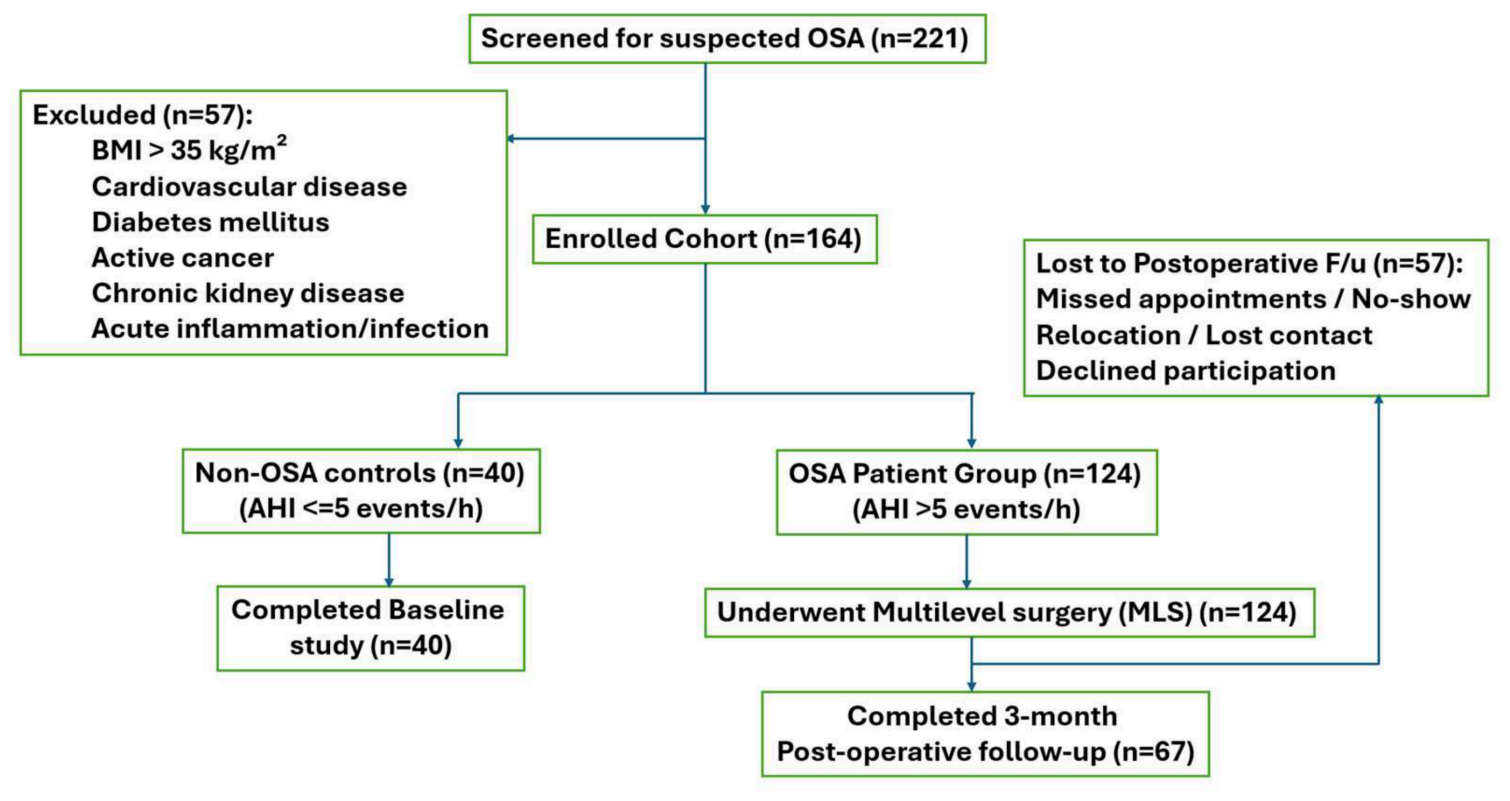
Participant recruitment and follow-up flow chart diagram.

The study cohort comprised 164 individuals, of whom 124 were patients with OSA and 40 were non-OSA controls (Figure 1). Baseline demographic and clinical characteristics were obtained from all participants, including age, sex, anthropometric measures, blood pressure, and polysomnographic parameters. Subsequent measurements were obtained for patients undergoing MLS to evaluate treatment efficacy. Eligible participants were adults aged 18–65 years who had been referred for evaluation of suspected OSA. Exclusion criteria involved prior history of cardiovascular disease, diabetes mellitus, active cancer, chronic kidney disease, or acute inflammatory or infectious conditions (Figure 1). Participants with severe obesity (body mass index [BMI] > 35) were excluded, as this condition represents a contraindication to OSA surgery. Individuals with BMI values below this threshold remained eligible for inclusion, given the strong association between obesity and OSA and its relevance to the study objectives. BMI was recorded for all participants and incorporated as a covariate in the statistical analyses to account for its potential confounding effect.

Fasting blood samples were collected from all participants at baseline using Vacutainer EDTA tubes (Becton, Dickinson and Company (BD), Franklin Lakes, NJ, USA) containing aprotinin. The samples were centrifuged at 400× *g* for 10 min at room temperature to separate plasma. The plasma fraction was then aliquoted and stored at −80 °C until further analysis.

This study was conducted at Dasman Diabetes Institute (DDI) in Kuwait, following approval from the Ethical Review Committee (ERC), as abiding by the ethical guidelines outlined in the Declaration of Helsinki (KFAS, RA-2015-043, dated 3 June 2016, and second post progress report Approval No.: RA HM-2020-014, Date: 1 October 2023). Participants were recruited from the sleep clinic according to predefined eligibility criteria, and written informed consent was obtained prior to enrollment.

### 2.2. OSA Assessment and Surgery

The study population underwent diagnostic evaluation using Type I PSG conducted in a Level I sleep laboratory to determine the presence of sleep apnea. The overnight assessment records multiple biophysiological signals, formulating a comprehensive characterization of sleep architecture. Specifically, Type I PSG monitors blood oxygen saturation, respiratory airflow and effort, electroencephalographic activity (EEG), electrooculographic activity (EOG), cardiac rhythm (ECG), and skeletal muscle activity (EMG). These measurements are used to calculate the apnea–hypopnea index (AHI). The apnea index (AI) represents the number of complete respiratory cessations lasting longer than 10 s per hour of sleep, whereas the hypopnea index (HI) reflects the number of partial airway obstruction events per hour. Diagnosis of OSA was established based on AHI values, with an AHI > 5 events/hour considered abnormal. OSA severity was classified according to standard thresholds: 5–15 events/h (mild), 15–30 events/h (moderate), and >30 events/h (severe) [1].

All patients undergoing surgical management received tonsillectomy, combined with additional procedures depending on the anatomical site of upper airway obstruction. Additional interventions included nasal surgery, palatal procedures, or base-of-tongue surgery, as indicated. MLS was performed in a single operative session by the same surgeon. While 57 participants of the initial cohort of 124 patients with OSA did not successfully complete the surgical postoperative follow-up after 3 months, 67 participants did. Multilevel sleep surgery was individualized according to each patient’s anatomical pattern of upper airway obstruction, as determined by comprehensive preoperative evaluation, including Drug-Induced Sleep Endoscopy (DISE). Surgical treatment targeted one or more levels of obstruction, including the nasal cavity, palate, oropharynx, tongue base, and hypopharynx, based on the identified sites of collapse. Consequently, most patients underwent a combination of procedures rather than a single operation. The most frequently performed procedures included soft palate surgery (barbed reposition pharyngoplasty, radiofrequency ablation, or a combination thereof) (n = 105), palatine tonsillectomy (n = 104), uvuloplasty (n = 65), septoplasty with inferior turbinate reduction (n = 62), adenoidectomy (n = 33), lateral pharyngeal wall procedures (n = 13), endoscopic sinus surgery involving the ethmoid, maxillary, and/or sphenoid sinuses (n = 12), endoscopic sinus surgery including the frontal sinus (n = 5), nasal polypectomy (n = 4), and lingual tonsillectomy (n = 1). As many patients underwent multiple procedures during the same operative session, these frequencies are not mutually exclusive. To assess for potential selection bias, a comprehensive baseline comparison was performed between the two groups (Supplementary Table S1). There were no statistically significant differences seen in terms of the major demographics, anthropometrics, metabolic indices, sleep architecture parameters (including baseline AHI, *p* = 0.807), or circulating biomarker profiles (including baseline IGFBP-7, *p* = 0.181) at the beginning of the study. This establishes that the investigated subgroup was highly representative of the broader baseline OSA cohort.

### 2.3. Measurement of Circulating Biomarkers

Plasma concentrations of IGFBP-7, together with IGFBP-1, IGFBP-2, IGFBP-3, IGFBP-4, TNF-α, IL-10, Orexin-A, and leptin, were quantified using a custom multiplex bead-based immunoassay (Luminex custom-made panel, Cat. #LXSAHM, R&D Systems, Newark, CA, USA) according to the manufacturer’s instructions. Data were acquired on a Luminex 200 system, and analyte concentrations were determined from standard curves using five-parameter logistic (5-PL) nonlinear regression with Bio-Plex Manager software (version 6.0; Bio-Rad, Hercules, CA, USA). Assay precision showed intra-assay coefficients of variation of 1.2–3.8% and inter-assay coefficients of variation of 2.8–5.2%.

Circulating levels of C-peptide and insulin were measured using enzyme-linked immunosorbent assays (ELISA) performed according to the manufacturer’s instructions (Mercodia AB, Sylveniusgatan, Sweden; Cat. #10-1136-01 and Cat. #10-1113-01, respectively). Absorbance for standards and samples was measured at 450 nm using a Synergy HTx microplate reader (BioTek, Agilent, Santa Clara, CA, USA). Concentrations were calculated from standard curves using Gen5TM 2.0 software. The intra-assay coefficient of variation ranged from 2.0 to 5.0%, while the inter-assay coefficient of variation was <10%.

### 2.4. Statistical Analysis

Continuous variables were first assessed for normality using the Shapiro–Wilk test in conjunction with visual inspection of distribution histograms and Q-Q plots. Variables exhibiting non-normal distributions were transformed using a rank-based inverse normal transformation (inverse distribution function transformation), in which ranked empirical observations were mapped to standard-normal quantiles. This normalization procedure was explicitly performed to satisfy the underlying distributional assumptions required for subsequent parametric inferential testing. To facilitate direct clinical interpretation, all descriptive data presented in text and tables are reported in their original, untransformed measurement units as mean ± standard error of the mean (SEM) or mean ± standard deviation (SD), as specified.

Between-group comparisons for normally distributed or normalized continuous variables were performed using an independent Student’s *t*-test. Within-group longitudinal differences between baseline and the 3-month follow-up in the OSA cohort were evaluated using a paired Student’s *t*-test. Categorical data, including gender distributions between the OSA and non-OSA groups, were assessed using the Chi-square (χ^2^) test. Associations between circulating insulin-like growth factor-binding protein-7 (IGFBP-7) levels and clinical, anthropometric, and biochemical parameters were examined using Pearson correlation analysis.

To address potential overfitting and optimism bias inherent in deriving and testing predictive algorithms within identical data configurations, a robust split-sample internal validation protocol was implemented. The study population was randomly partitioned into a derivation (training) cohort comprising approximately 80% of the total sample, and an independent validation (testing) cohort comprising the remaining 20% holdout sample using a random Bernoulli distribution (*p* = 0.80).

A multivariable binary logistic regression model was constructed exclusively within the derivation cohort to identify independent clinical predictors of OSA status (defined as an Apnea–Hypopnea Index [AHI] > 5 events/hour) versus non-OSA controls (≤5 events/hour). The candidate biomarkers, IGFBP-7 and IGFBP-4, were entered as continuous variables in their original measurement units (pg/mL); thus, the calculated odds ratios (ORs) represent the change in the odds of OSA presence per 1 pg/mL increase in biomarker concentration. Covariates included in the multivariable model were selected a priori based on established clinical relevance, peer-reviewed recommendations, and univariate statistical significance. The final adjusted model simultaneously accounted for age, sex, BMI, total cholesterol, triglycerides, fasting insulin levels, and nocturnal O_2_ saturation parameters to evaluate the independent predictive risk associated with circulating plasma IGFBP-7 and IGFBP-4 levels.

The resulting regression equation coefficients from the training phase were then frozen and applied to the independent validation cohort to generate unbiased, predicted probability scores for each remaining participant. The diagnostic and discriminative performance of these predicted probabilities in the unseen validation cohort was evaluated using the Receiver Operating Characteristic (ROC) curve analysis. The unbiased Area Under the Curve (AUC) was calculated along with its asymptotic 95% confidence interval (CI). The optimal classification threshold was determined using coordinate optimization, prioritizing a decision boundary that maximizes sensitivity while minimizing the false-positive rate (1-specificity) for clinical utility. All statistical workflows were executed using IBM SPSS Statistics for Windows, version 25.0 (IBM SPSS Inc., Armonk, NY, USA). A two-tailed *p*-value < 0.05 was considered statistically significant.

#### Sample Power

Population size was determined by using power statistics to ensure an adequate sample size. According to our objectives, the primary endpoint is the difference in circulation IGFBP-7 levels between patients with OSA and the control group. The analysis assumed a two-tailed a = 0.05, a moderate effect size (Cohen’s d = 0.5), and 80% power; thus, the minimum required sample size was 64 participants (32 per group). To achieve 90% power, a total of 86 participants would be required. The final study cohort included patients with OSA (n = 124) and controls (n = 40), exceeding the calculated minimum requirements and providing adequate statistical power to detect the hypothesized effect.

## 3. Results

### 3.1. Study Cohort and Baseline Features

The study population comprised 164 participants, where men comprised 84% of the cohort. The demographic, clinical, metabolic, and sleep-related characteristics of the study population are summarized in Table 1. Our data show no significant differences between the OSA and control groups with respect to baseline clinical characteristics, including age, sex distribution, height, pulse rate, systolic and diastolic blood pressure, lipid profile, glucose, HbA1c and whole blood counts (all *p* > 0.05), indicating that the groups were broadly comparable. HDL-C levels were an exception, with higher levels in the CTRL group (i.e., people with no OSA) than in those with OSA, a difference that reached statistical significance.

**Figure 2:**
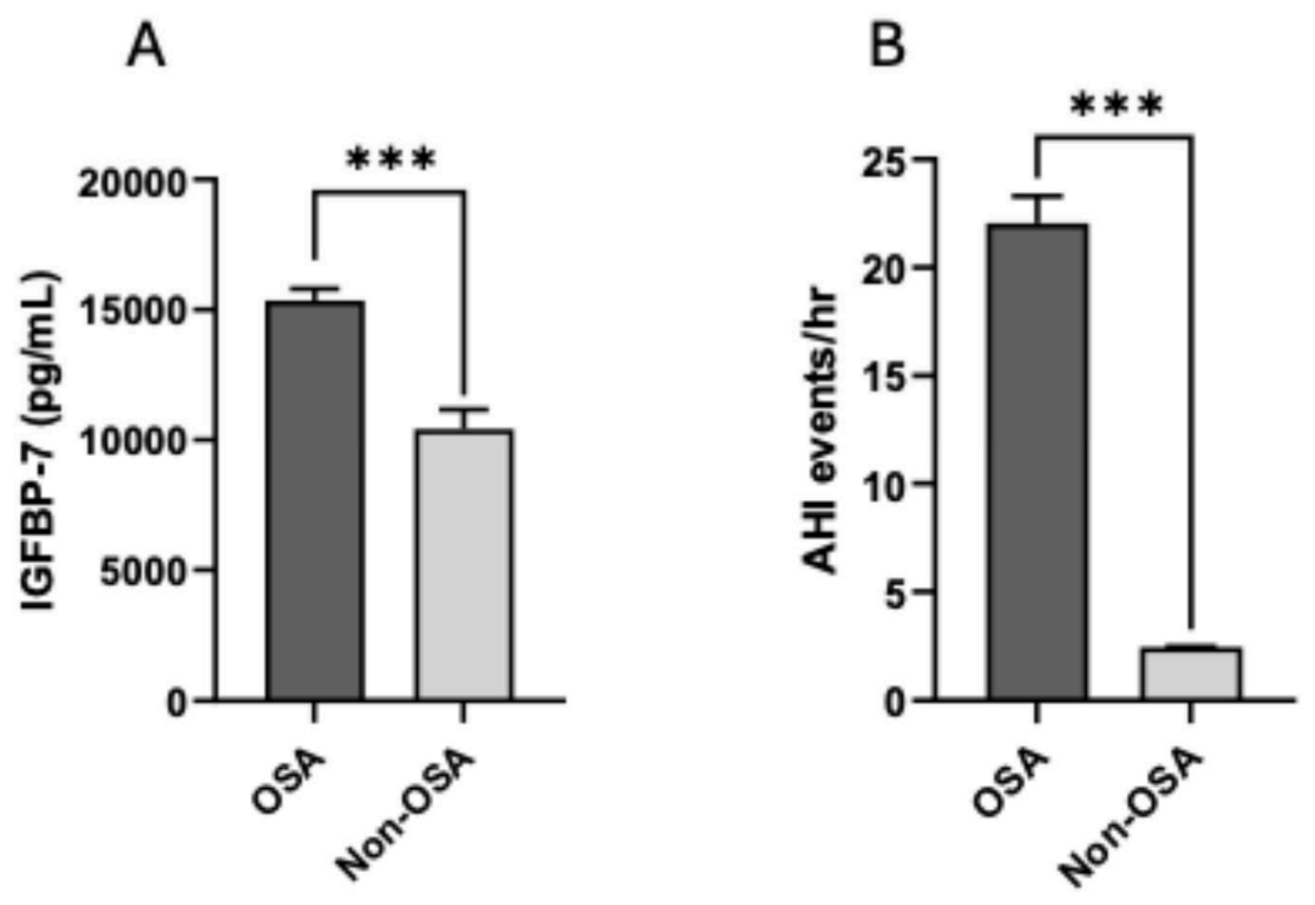
Circulating IGFBP-7 levels and AHI in OSA and Non-OSA groups. (**A**) Baseline IGFBP-7 levels were significantly higher in individuals with OSA compared to controls. (**B**) AHI was significantly higher in the OSA group compared to the non-OSA group. Data are Mean ± standard Error of the Mean (SEM). *** *p* < 0.001.

Significant differences in body mass index (BMI) were observed between the OSA and control groups (*p* < 0.001), reflecting the well-established association between obesity and increased OSA risk. Additionally, people with OSA had significantly higher AHI values (22.06 ± 1.25 events/hour) than controls (2.45 ± 0.22 events/hour; *p* < 0.001; Figure 2B). Consistent with this finding, Epworth Sleepiness Scale (ESS) scores were also significantly higher in the OSA group (*p* < 0.001), indicating greater daytime sleepiness. Several metabolic markers differed significantly between the OSA and control groups, including C-peptide and insulin levels, which were higher in participants with OSA (*p* = 0.006 and *p* = 0.025, respectively), suggesting altered insulin regulation.

Our data show a selective alteration in the IGFBP superfamily, with higher levels of circulating IGFBP-4 and IGFBP-7 in the OSA group (*p* = 0.006 and *p* < 0.001, respectively). while other proteins, such as IGFBP-1, IGFBP-2, and IGFBP-3, show no difference (Table 1). Inflammatory markers such as IL-10 and Orexin-A were significantly elevated in participants with OSA, whereas TNF-α and leptin levels did not differ between groups.

### 3.2. Increased IGFBP-7 and Its Association with OSA Severity

**Figure 3:**
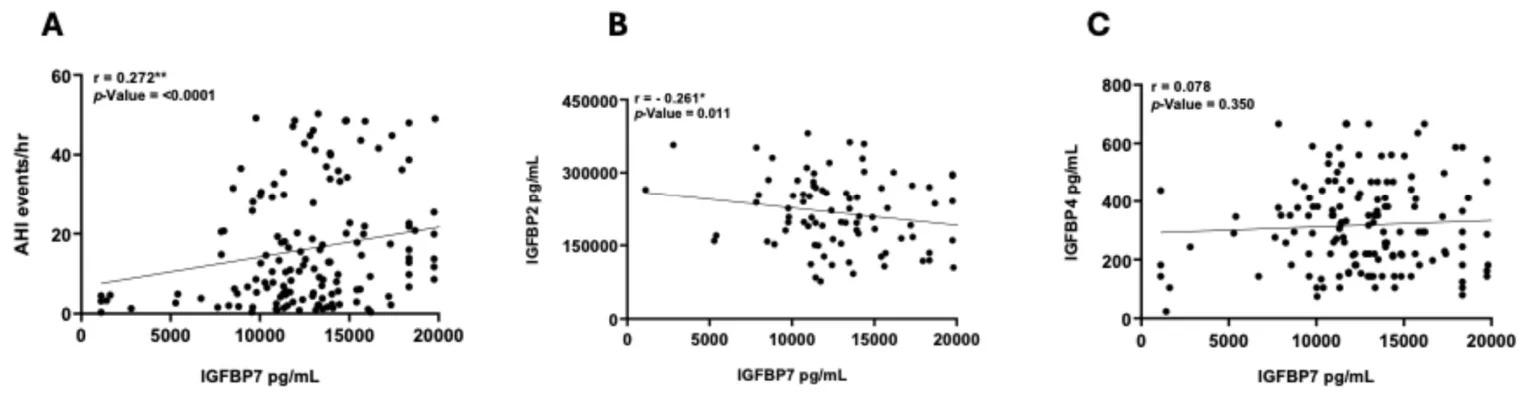
Pearson correlation analysis between circulating IGFBP-7 and (**A**) apnea–hypopnea index (AHI) that demonstrates a significant positive correlation, (**B**) IGFBP2 displaying a significant negative correlation, (**C**) IGFBP-4 displaying no association. * *p* < 0.05; ** *p* < 0.01.

Our study population shows a significant increase in circulating IGFBP-7 levels in people with OSA (15,358.70 ± 453.19 pg/mL) compared with control participants (10,429.70 ± 747.62, *p* < 0.001 pg/mL; Figure 2A). Additionally, IGFBP-7 showed a significant positive correlation with AHI (r = 0.272, *p* < 0.001; Figure 3A), suggesting an association between OSA and higher circulating IGFBP-7 (Table 2). Similar positive correlations were observed between IGFBP-7 and AI (r = 0.212, *p* = 0.006) and HI (r = 0.212, *p* = 0.006). BMI showed a modest but significant positive correlation with IGFBP-7 (r = 0.175, *p* = 0.026), implicating a potential link between obesity and IGFBP-7. Among the IGFBP superfamily, IGFBP-2 showed a significant inverse correlation with IGFBP-7 (r = −0.261, *p* = 0.011; Figure 3B), whereas IGFBP-4 showed no correlation with IGFBP-7 levels (Figure 3C). Interestingly, IGFBP-7 showed a significant positive correlation with c-peptide (r = 0.219, *p* = 0.005).

### 3.3. Post-MLS Surgical Intervention OSA-Relief Is Associated with Lower IGFBP-7 Levels

**Figure 4:**
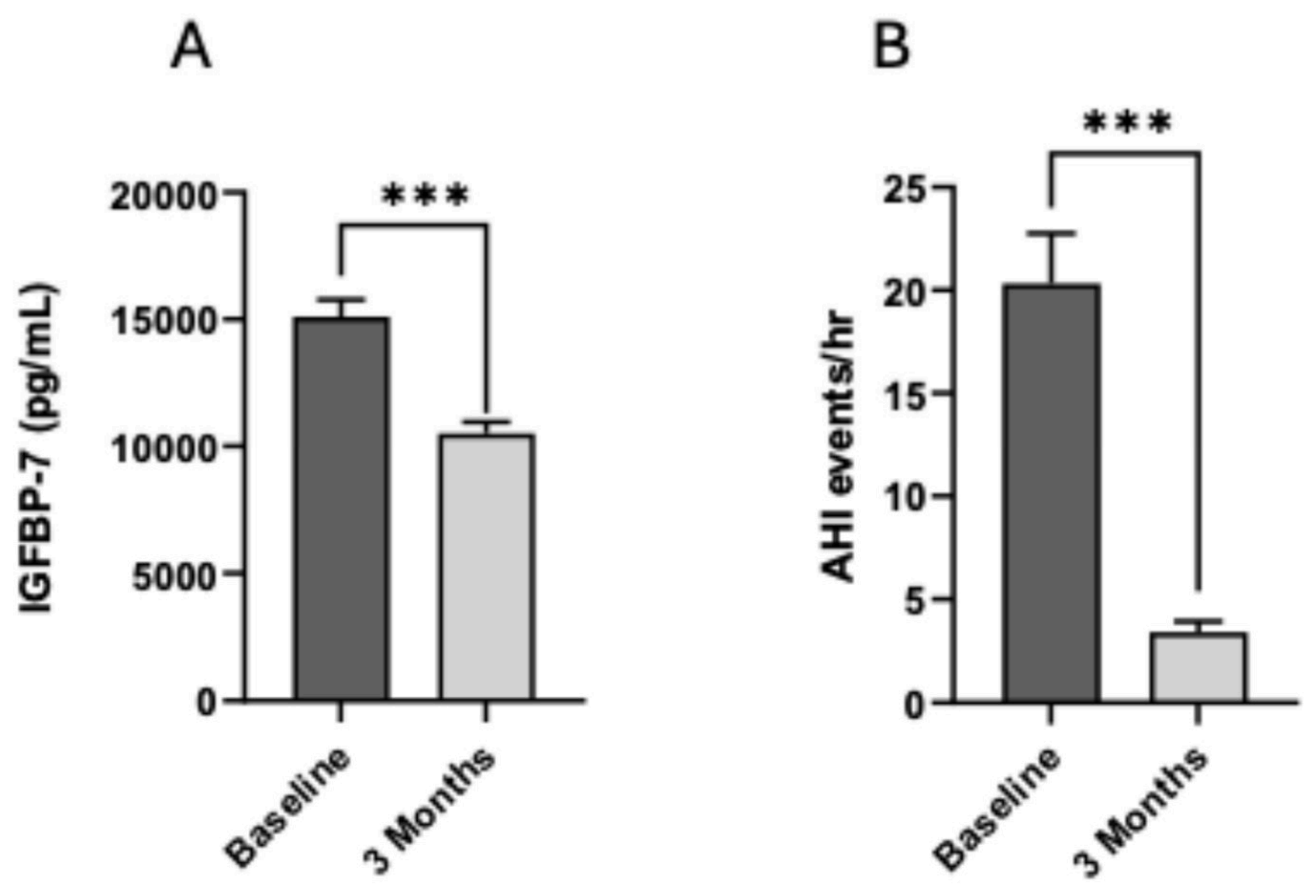
Circulating IGFBP-7 levels and AHI in OSA group following multi-level surgery. (**A**) A significant decrease in IGFBP-7 levels after 3 months compared to baseline (**B**) MLS as a corrective intervention for OSA resulted in a significant reduction in AHI (*** *p* < 0.001). Data are Mean ± standard error of the mean (SEM).

To further examine the relationship between OSA and circulating IGFBP-7 levels, we quantified circulating IGFBP-7 levels three months following surgical intervention in a subset of the OSA group (n = 67). Additionally, comparing baseline clinical and biochemical parameters with those three months post-surgery showed significant improvements in sleep-related indices (Table 3). Epworth Sleepiness Scale (ESS) scores were markedly reduced (*p* < 0.001). Similarly, significant reductions were observed in other indices, including AI, HI, and AHI (*p* = 0.013, *p* < 0.001, *p* < 0.001, respectively; Figure 4B), indicating improved OSA severity following surgical intervention.

Furthermore, we detected a significant decline in circulating IGFBP-7 levels three months post-MLS (15,102.57 ± 679.17 vs. 10,516.42 ± 446.93 pg/mL, *p* < 0.001; Figure 4A). Additionally, IGFBP-4 levels decreased significantly following MLS surgery (*p* = 0.025). Other clinical and biochemical parameters did not show any significant change from baseline at the three-month follow-up.

### 3.4. A Potential OSA–IGFBP-4 and IGFBP-7 Link

We conducted a comprehensive binary logistic regression model to ascertain if the clinical correlations between circulating IGFBP-4 and IGFBP-7 levels and OSA are independent of anthropometric, lipid, glycemic, and hypoxic variables (Table 4). The model concurrently controlled for the whole panel of clinical confounders raised during peer review, specifically including age, sex, BMI, total cholesterol, triglycerides, nocturnal O_2_ saturation parameters, and fasting insulin levels.

Notably, even with comprehensive multivariable adjustment and simultaneous evaluation, both plasma biomarkers maintained strong, statistically significant independent associations with OSA status. After multivariable adjustment, circulating IGFBP-7 remained independently associated with OSA status (OR = 1.61, 95% CI: 1.13–2.29, *p* = 0.009), indicating a 61% increase in the odds of OSA for each 1000 pg/mL increase in IGFBP-7. IGFBP-4 was also independently associated with OSA (OR = 1.10, 95% CI: 1.03–1.17, *p* = 0.003), corresponding to a 10% increase in the odds of OSA for each 1000 pg/mL increase. In contrast, traditional metabolic and adiposity confounders such as BMI (*p* = 0.144), fasting insulin (*p* = 0.919), and serum triglycerides (*p* = 0.887) demonstrated no independent predictive value in the fully adjusted model. These data indicate that the association between circulating insulin-like growth factor-binding proteins and obstructive sleep apnea remains significant after adjustment for key components of obesity and metabolic syndrome, supporting the robustness of the observed association while acknowledging the possibility of residual confounding inherent to observational studies.

Our analysis showed no correlation between IGFBP-4 and IGFBP-7. The multicollinearity test revealed a low variance inflation factor (VIF = 1.006), indicating no collinearity between the variables. Consequently, both IGFBP-4 and IGFBP-7 were included in the multivariable logistic regression model to assess their independent associations with OSA.

### 3.5. Receiver Operating Characteristic Curve Analysis of IGFBP-7 to Indicate OSA

**Figure 5:**
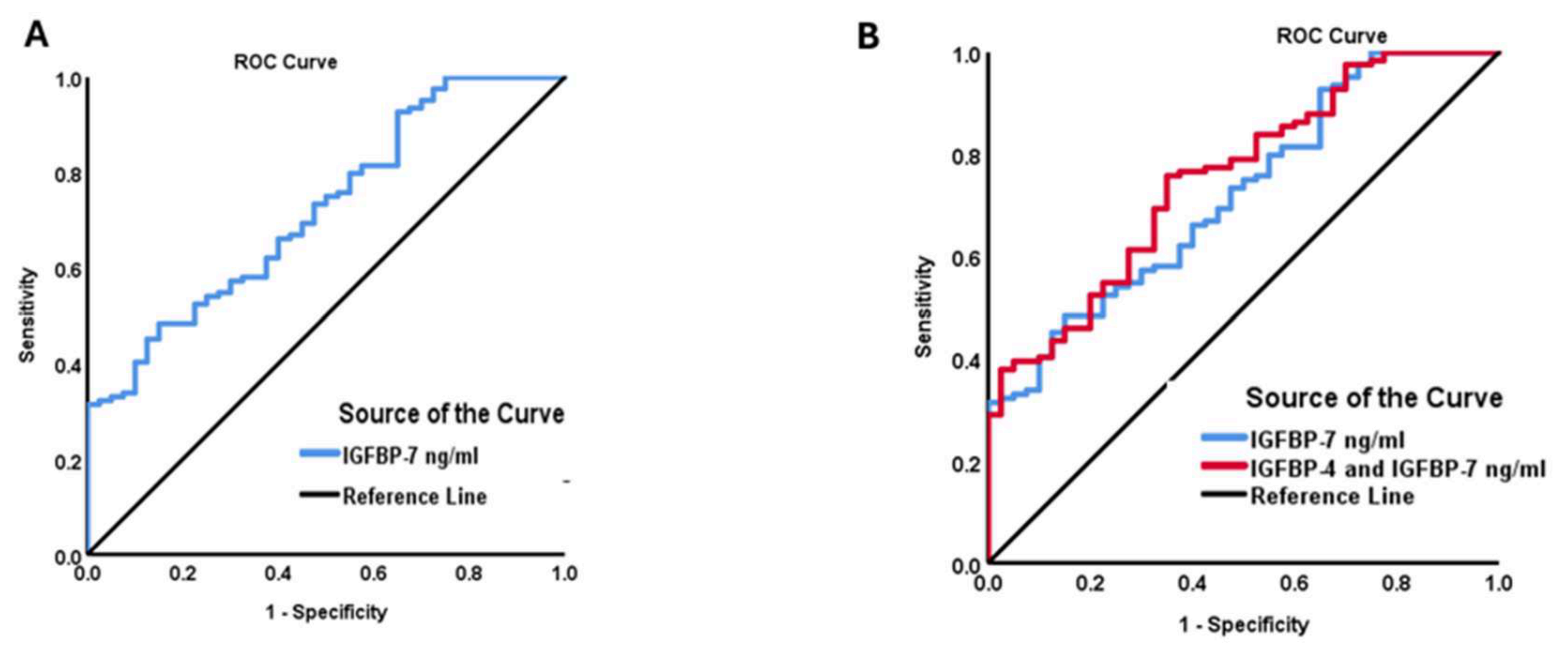
Results Receiver operating characteristic (ROC) analysis evaluating the diagnostic performance of plasma biomarkers for OSA. (**A**) IGFBP-7 alone demonstrated moderate diagnostic performance with an area under the curve (AUC) of 0.722 (95% CI: 0.636–0.808; sensitivity: 73%, specificity: 84%). (**B**) The combined model of IGFBP-7 and IGFBP-4 improved discrimination, yielding an AUC of 0.755 (95% CI: 0.673–0.837; *p* < 0.001).

To assess the ability of circulating IGFBP-7 to discriminate between people with OSA and controls, and to determine an optimal cut-off value, we performed receiver operating characteristic (ROC) curve analysis (Figure 5). IGFBP-7 showed moderate discriminatory performance, with a cut-off value of 14,003.21 pg/mL and an area under the curve (AUC) of 0.722 (95% CI: 0.636–0.808; Figure 5A). The combined model, including IGFBP-7 and IGFBP-4, showed a slight improvement in discrimination, yielding an AUC of 0.755 (95% CI: 0.673–0.837; Figure 5B). At the optimal threshold, IGFBP-7 identified individuals with OSA with 73% sensitivity and 84% specificity.

## 4. Discussion

In this report, we introduce IGFBP-7 as a potential biomarker associated with OSA. We observed a significant increase in circulating IGFBP-7 levels in individuals with OSA compared to controls. The link between OSA and IGFBP-7 was further supported by a positive correlation between elevated IGFBP-7 levels and OSA, indicated by a positive correlation with AHI. Additionally, patients with OSA who underwent MLS treatment to alleviate symptoms showed a significant reduction in circulating IGFBP-7. This supports the connection between IGFBP-7 and OSA and implicates that the modest association between IGFBP-7 and OSA severity could support an exploratory role for IGFBP-7 in a multi-marker approach. Overall, these findings suggest a potential role for IGFBP-7 in a multi-marker panel for assessing disease presence and treatment efficacy in patients with OSA. It is not proposed as a replacement for polysomnography.

IGFBP-7 is a secreted protein within the IGFBP superfamily, characterized by its relatively low affinity for IGF ligands and a higher affinity for insulin [14,25]. It is widely expressed across multiple tissues, particularly in the vascular endothelium and respiratory epithelium, which are tissues pertinent to the pathophysiology of OSA [15,26]. Mechanistically, levels of IGFPB-7 mRNA are reported to increase in glioma cells subjected to hypoxia, which is mainly dependent on inositol-requiring enzyme 1 (IRE-1), a mediator of endoplasmic reticulum stress [27]. Although a hypoxia IGFBP-7-dedicated study is lacking, the hypoxia response element (HRE)-mediated mechanism is established for IGFBP-1, and HIF-1α along with associated transcription factors are known to modulate various IGFBPs and presumably this extends to IGFBP-7 but the direct mechanistic chain remains less characterized [28]. The elevation in plasma IGFBP-7 has been significantly associated with heart failure [29]. Thus, the relationship between IGFBP-7 and OSA can be elucidated through its emerging biological functions. IGFBP-7 is notably associated with cellular senescence and elements of the senescence-associated secretory phenotype (SASP) [18,30,31], which is of particular importance in OSA. Given that oxidative stress, a recognized characteristic of OSA, significantly contributes to cellular senescence, this implies a potential mechanistic link between OSA-induced stress and IGFBP-7 expression [5,32,33,34]. The increased levels of circulating IGFBP-7 in people with OSA in the current study may be related to chronic intermittent hypoxia, which induces oxidative stress and inflammatory responses [5].

In addition to serving as an indicator of cellular stress, IGFBP-7 may also reflect metabolic dysregulation in OSA. The condition is often associated with insulin resistance and impaired glucose homeostasis, even in individuals without overt diabetes [3], corroborated by elevated insulin and C-peptide levels observed in the present study. IGFBP-7 may indicate disturbances in relevant metabolic processes through its interaction with insulin and IGF signalling pathways [25]. The positive correlation between C-peptide and IGFBP-7 corroborates a previous report [35] and suggests a potential association with altered insulin secretion and related metabolic processes [36]. Furthermore, IGFBP-7 has demonstrated clinical relevance as a biomarker in conditions characterized by cellular stress and tissue damage, such as acute kidney injury and cardiovascular disease, which share overlapping pathophysiological features with OSA [36], thereby reinforcing its prospective utility in this context.

In a previous study with a smaller cohort, [11], we found a non-significant elevation in circulating IGFBP-7 levels compared with the control. The current study involved a larger and more rigorously matched cohort that demonstrated a statistically significant rise in circulating IGFBP-7 in people with OSA compared to the non-OSA group. While the direction of effect is consistent across both studies (i.e., higher IGFBP-7 levels in people with OSA), there is a considerable difference in the magnitude that might be attributed to various cohort-dependent factors. Using Pearson correlation analysis, we observed positive correlations between IGFBP-7 and indices of OSA, including AHI, AI and HI, further implicating IGFBP-7 as a potential disease-related biomarker. A modest correlation between IGFBP-7 and BMI was also observed in both the OSA and non-OSA groups, consistent with a previous report [37], thereby reflecting an association with adiposity and its metabolic consequences. However, IGFBP-7 remained independently associated with OSA after adjustment for BMI and other covariates, implicating that the relationship with OSA might extend beyond obesity alone.

Moreover, our data indicated a significant increase in IGFBP-4 levels among individuals with OSA, corroborating a prior report [11]. The elevation in IGFBP-4 was inversely related to IGFBP-2 but not associated with IGFBP-7, indicating differential regulation within the IGFBP family. This pattern suggests that these proteins represent different but partially overlapping biological pathways, emphasizing a unique role of IGFBP-7 [25]. There was a significant reduction in circulating IGFBP-7 levels following multilevel surgery, coinciding with improvements in AHI score, underscoring the potential significance of IGFBP-7 in OSA. Although the direction of the association between circulating IGFBP-7 and OSA was consistent across our previous and current studies, differences in the magnitude of the observed effect may reflect variations in cohort characteristics, disease severity distribution, or analytical variability. Therefore, IGFBP-7 should be considered a promising candidate biomarker for inclusion in multi-marker panels for OSA diagnosis and treatment monitoring. Independent validation in larger, multicenter cohorts is required to confirm its reproducibility and clinical utility.

The diagnostic efficacy of IGFBP-7 demonstrated in this study underscores its potential clinical significance in OSA. Despite IGFBP-7 exhibiting only moderate discriminative capacity (AUC ~0.72), with acceptable sensitivity and specificity (73% and 84%, respectively), this performance level aligns with the complex and heterogeneous characteristics of OSA, in which no single biomarker is likely adequate for diagnosis [8]. This performance level is also comparable to that of other circulating biomarkers in OSA, which often exhibit intermediate diagnostic accuracy rather than strong discriminatory power [8,12,38]. IGFBP-7 may offer additional value within a multimodal diagnostic paradigm rather than functioning as an independent test. The slight performance enhancement observed with the combination of IGFBP-7 and IGFBP-4 indicates that including biomarkers that reflect complementary biological processes may yield a more precise depiction of disease complexity and improve disease detection. The reported AUC values are classified as fair to moderate according to conventional standards. This allows us to interpret our data as hypothesis-generating and as a basis for future studies that combine IGFBP-7 with clinical variables, rather than data ready for clinical utility.

Our study encompasses several limitations that warrant careful consideration. This is a single-centre study with a predominantly male cohort, which may limit the generalizability of the findings to more diverse populations. Secondly, while the OSA and control groups were generally equivalent across all clinical parameters, individuals with OSA exhibited a higher BMI, and a modest correlation between BMI and IGFBP-7 was noted. Although IGFBP-7 remained independently associated with OSA after controlling for BMI, residual confounding effects cannot be completely ruled out. Major comorbidities were excluded; nonetheless, IGFBP-7 is not specific to OSA and may reflect systemic cellular stress associated with multiple pathological conditions. Therefore, IGFBP-7 should be considered a biomarker associated with OSA rather than a disease-specific marker, supporting the use of multi-marker panels to improve diagnostic specificity. A significant weakness in our report is the lack of prior mechanistic studies using direct hypoxic burden markers (e.g., oxygen desaturation index, time spent below 90% SpO_2_).

An additional limitation of this study is the relatively high postoperative attrition rate, with only 67 of 124 patients completing the 3-month follow-up. However, baseline demographic, clinical, and biochemical characteristics, including OSA severity indices and circulating IGFBP-7 levels, were comparable between participants who completed follow-up and those who did not (Supplementary Table S1), suggesting that attrition was unlikely to have introduced substantial selection bias. Nevertheless, the reduced follow-up sample size may have limited the statistical power of the longitudinal analyses, warranting confirmation in larger prospective studies. Finally, mechanistic investigations are beyond the scope of this study; therefore, future research should incorporate mechanistic analysis to enhance our comprehension of IGFBP-7 activity and significance. Future research should address these limitations by validating findings in larger, multicenter cohorts, including IGFBP-7 in multi-marker panels to improve clinical utility, and by conducting mechanistic studies to clarify IGFBP-7’s possible role in hypoxia- and metabolism-related pathways.

## 5. Conclusions

This study reports a significant increase in plasma IGFBP-7 levels in people with OSA. This increase was followed by a significant decline after a corrective surgical procedure (i.e., MLS) to alleviate OSA symptoms, reinforcing a potential role for IGFBP-7 in a disease-related physiological changes. Nonetheless, the moderate diagnostic efficacy and the lack of mechanistic data caution against interpreting it as a standalone or novel clinical tool. At present, IGFBP-7 appears best suited as one component of a multi-marker panel, potentially working in conjunction with other bioindicators of cellular stress or metabolic disturbance. The potential clinical relevance of IGFBP-7 remains preliminary, and effective translation and validation of this hypothesized role require rigorous validation in larger, prospective multicenter cohorts before the consideration of any practical application.

## Figures and Tables

**Table 1:** Clinical and Biochemical characteristics of participants.

Variables	OSA (n = 124)	Non-OSA (n = 40)	*p*-Value
Age in Years	43.55 ± 11.18	44.75 ± 11.19	0.557
Gender M/F	105/19	33/7	0.745
ESS	13.32 ± 5.93	4.00 ± 4.40	<0.001
Weight Kg	91.23 ± 17.98	82.68 ± 15.80	0.005
Height cm	171.23 ± 8.76	170.99 ± 9.66	0.889
Body Mass Index (kg/m^2^)	31.04 ± 5.09	27.94 ± 4.26	<0.001
Pulse	74.94 ± 10.92	74.15 ± 10.93	0.698
Systolic Blood Pressure mmHg	126.21 ± 13.13	123.74 ± 11.57	0.270
Diastolic Blood Pressure mmHg	73.80 ± 9.14	75.44 ± 8.02	0.292
AI events/h	6.37 ± 8.41	0.77 ± 0.89	<0.001
HI events/h	15.69 ± 10.90	1.68 ± 1.45	<0.001
AHI events/h	22.06 ± 13.92	2.45 ± 1.39	<0.001
Total Cholesterol mmol/L	5.00 ± 1.10	4.79 ± 1.01	0.275
HDL-C mmol/L	1.13 ± 0.31	1.27 ± 0.29	0.012
LDL-C mmol/L	3.23 ± 1.01	2.98 ± 0.94	0.162
Triglycerides mmol/L	1.50 ± 0.98	1.33 ± 0.81	0.260
Glucose mmol/L	5.89 ± 1.50	5.78 ± 1.61	0.716
HBA1C %	5.87 ± 1.07	5.77 ± 1.10	0.620
Whole Blood Counts 10^9^/L	7.03 ± 1.97	6.53 ± 1.72	0.126
C-Peptide pmol/L	3278.44 ± 1560.98	2528.19 ± 1391.25	0.006
Insulin U/L	10.10 ± 6.44	7.93 ± 4.72	0.025
IGFBP-1 pg/mL	11,989.99 ± 15,119.64	13,837.68 ± 13,593.48	0.471
IGFBP-2 pg/mL	205,181.75 ± 70,909.20	237,645.57 ± 79,910.06	0.126
IGFBP-3 pg/mL	655,055.12 ± 194,073.17	680,842.28 ± 215,939.59	0.504
IGFBP-4 ng/mL	337.13 ± 159.92	270.72 ± 118.84	0.006
IGFBP-7 pg/mL	15,358.70 ± 5046.52	10,429.70 ± 4728.37	<0.001
O_2_ saturation %	96.36 ± 2.18	96.78 ± 1.45	0.353
IL-10 pg/mL	1.57 ± 1.33	0.85 ± 0.50	<0.001
Orexin-A pg/mL	79.57 ± 19.82	68.00 ± 11.63	<0.001
TNF-α pg/mL	0.52 ± 1.10	0.64 ± 1.30	0.713
Leptin pg/mL	12,821.71 ± 9915.33	11,594.01 ± 10,479.83	0.522

Data are Mean ± standard deviation (SD), ESS: Epworth sleepiness scale; AHI: Apnea–hypopnea index; AI: Apnea index; HI: Hypopnea index.

**Table 2:** Pearson correlations of IGFBP-7 with clinical biochemical parameters in the full cohort.

Variables	*r*	*p* Value
Age in Years	0.039	0.620
ESS	0.206	0.274
Weight Kg	0.109	0.165
Height cm	−0.019	0.808
Body Mass Index (kg/m^2^)	0.175 *	0.026
Pulse	0.049	0.541
Systolic Blood Pressure mmHg	0.020	0.802
Diastolic Blood Pressure mmHg	−0.063	0.435
AI events/h	0.212 **	0.006
HI events/h	0.212 **	0.006
AHI events/h	0.272 **	<0.001
Total Cholesterol mmol/L	−0.060	0.447
HDL-C mmol/L	−0.018	0.816
LDL-C mmol/L	−0.080	0.311
Triglycerides mmol/L	0.122	0.121
Glucose mmol/L	0.068	0.392
HBA1C %	0.090	0.257
WBC 10^9^/L	0.029	0.712
C-Peptide pmol/L	0.219 **	0.005
Insulin U/L	0.143	0.068
IGFBP-1 pg/mL	0.030	0.704
IGFBP-2 pg/mL	−0.261 *	0.011
IGFBP-3 pg/mL	0.074	0.350
IGFBP-4 pg/mL	0.078	0.324
O_2_ saturation %	−0.144	0.247
IL-10 pg/mL	−0.127	0.217
Orexin-A pg/mL	0.094	0.230
TNF-α pg/mL	−0.085	0.406
Leptin pg/mL	0.025	0.754

** *p* < 0.01; * *p* < 0.05.

**Table 3:** Comparison between pre- and post-intervention in OSA group (post 3 months).

Variables	Baseline (n = 67) Mean ± SD	Post 3 Months (n = 67) Mean ± SD	*p* Value
ESS	12.08 ± 6.11	2.83 ± 2.48	<0.001
Weight Kg	88.02 ± 17.42	87.75 ± 17.20	0.746
Height cm	170.55 ± 8.50	170.89 ± 8.68	0.141
Body Mass Index (kg/m^2^)	30.20 ± 4.82	29.52 ± 5.50	0.168
Pulse	76.29 ± 9.34	75.15 ± 9.40	0.353
Systolic Blood Pressure mmHg	126.46 ± 13.50	126.12 ± 11.57	0.834
Diastolic Blood Pressure mmHg	74.36 ± 9.52	75.56 ± 8.25	0.245
AI events/h	5.28 ± 8.30	1.28 ± 2.77	0.013
HI events/h	15.04 ± 10.51	4.07 ± 3.38	<0.001
AHI events/h	20.32 ± 13.78	3.41 ± 2.95	<0.001
Total Cholesterol mmol/L	5.00 ± 1.22	4.96 ± 1.09	0.715
HDL-C mmol/L	1.10 ± 0.27	1.12 ± 0.27	0.490
LDL-C mmol/L	3.21 ± 1.12	3.17 ± 1.09	0.575
Triglycerides mmol/L	1.58 ± 1.09	1.51 ± 0.86	0.561
Glucose mmol/L	5.85 ± 1.21	5.76 ± 1.21	0.343
HBA1C %	5.77 ± 0.89	5.75 ± 0.82	0.798
Whole Blood Count 10^9^/L	7.08 ± 2.01	6.58 ± 1.66	0.002
C-Peptide pmol/L	3374.88 ± 1440.62	3445.49 ± 1483.57	0.656
Insulin U/L	10.04 ± 5.84	10.62 ± 6.94	0.428
IGFBP-1 pg/mL	12,085.96 ± 17,655.43	13,761.20 ± 15,235.13	0.519
IGFBP-2 pg/mL	196,307.93 ± 69,968.71	183,385.83 ± 74,534.68	0.296
IGFBP-3 pg/mL	643,563.48 ± 175,983.13	628,521.81 ± 177,288.62	0.473
IGFBP-4 ng/mL	404.91 ± 139.20	343.76 ± 166.21	0.025
IGFBP-7 pg/mL	15,102.57 ± 5390.74	10,516.42 ± 3547.37	<0.001
IL-10 pg/mL	1.32 ± 1.13	0.79 ± 1.32	0.088
Orexin-A pg/mL	77.44 ± 18.06	74.34 ± 14.95	0.341
TNF-α pg/mL	0.27 ± 0.57	0.29 ± 0.87	0.870
Leptin pg/mL	12,406.96 ± 9295.89	10,884.68 ± 7502.51	0.049

Data represented in Mean ± SD.

**Table 4:** Multivariable Logistic Regression Analysis evaluating the independent associations of plasma biomarkers with OSA status.

Model	Independent Variable	β (SE)	OR (95% CI)	*p*-Value
Dependent variable: OSA Status	IGFBP-7, per 1000 pg/mL increase	0.47 (0.18)	1.61 (1.13–2.29)	0.009
	IGFBP-4, per 1000 pg/mL increase	0.10 (0.03)	1.10 (1.03–1.17)	0.003
	Age (years)	−0.12 (0.05)	0.89 (0.81–0.97)	0.009
	Gender (male)	−0.08 (1.00)	0.92 (0.13–6.53)	0.933
	BMI (kg/m^2^)	0.17 (0.12)	1.19 (0.94–1.50)	0.144
	Total Cholesterol (mmol/L)	0.55 (0.50)	1.74 (0.65–4.62)	0.270
	Triglycerides (mmol/L)	−0.08 (0.55)	0.93 (0.32–2.71)	0.887
	Insulin (U/L)	−0.01 (0.11)	0.98 (0.80–1.22)	0.919
	O_2_ saturation (%)	−0.29 (0.28)	0.75 (0.43–1.30)	0.300

Data are presented as β coefficients (standard errors) and odds ratios (95% confidence intervals). IGFBP-7 and IGFBP-4 concentrations were expressed in pg/mL and scaled by dividing each value by 1000 before inclusion in the logistic regression model. Accordingly, the reported odds ratios represent the change in the odds of OSA associated with each 1000 pg/mL increase in biomarker concentration.

## Data Availability

Data will only be shared upon request from the corresponding author due to unpublished data and ethical restrictions by the institute.

## Supplementary Materials

The following supporting information can be downloaded at https://www.mdpi.com/article/10.3390/biomedicines14081780/s1. Table S1: Clinical and Biochemical characteristics of participants who completed the post-operative follow-up and patients who failed to complete the follow-up visit.
