# Supplementary material for: Circulating IGFBP-7 in Obstructive Sleep Apnea: A Single-Cohort Preliminary Assessment of Its Diagnostic Performance and Post-Treatment Dynamics
Source: Biomedicines. 2026 Aug 7;14(8):1780. doi: 10.3390/biomedicines14081780 (PMC13510262; doi:10.3390/biomedicines14081780)
Supplement: Supplementary file 1 [file biomedicines-14-01780-s001.zip › biomedicines-4379911-supplementary.pdf]

Supplementary Table S1

Variables	Completed 3-month postoperative follow-up (n=67) Mean ± SEM	Did not complete postoperative follow-up (n=57) Mean ± SEM	P value
Age in years	41.87 ± 1.34	45.11 ± 1.51	0.110
Gender M/F	57/10	46/11	0.632
Weight KGS	88.02 ± 2.18	87.13 ± 2.11	0.772
Height CMS	170.55 ± 1.05	168.52 ± 1.17	0.198
Body Mass Index (kg/m^2)	30.20 ± 0.62	28.62 ± 0.65	0.082
Pulse	76.29 ± 1.22	77.23 ± 1.19	0.586
Systolic Blood Pressure mmHg	126.46 ± 1.76	125.33 ± 1.89	0.663
Diastolic Blood Pressure	74.36 ± 1.24	76.47 ± 1.49	0.275
AI events/h	5.28 ± 1.47	5.22 ± 1.33	0.976
HI events/h	15.04 ± 1.86	14.02 ± 1.77	0.695
AHI events/h	20.32 ± 2.44	21.11 ± 2.01	0.807
Total Cholesterol mmol/l	5.00 ± 0.15	5.23 ± 0.23	0.391
HDL-C mmol/l	1.10 ± 0.03	1.12 ± 0.03	0.640
LDL-C mmol/l	3.21 ± 0.14	3.47 ± 0.23	0.321
Triglycerides mmol/l	1.58 ± 0.13	1.72 ± 0.44	0.745
Glucose mmol/l	5.85 ± 0.15	5.96 ± 0.17	0.628
HBA1C %	5.77 ± 0.11	5.93 ± 0.16	0.401
Whole Blood Counts 10^9/L	7.08 ± 0.25	7.47 ± 0.52	0.480
C-Peptide pmol/L	3374.88 ± 194.25	3146.13 ± 148.23	0.364
Insulin U/L	10.04 ± 0.79	11.23 ± 0.47	0.218
IGFBP1 ng/ml	12085.96 ±	12247.01 ± 1880.53	0.957
IGFBP2 pg/ml	196307.93 ±	172267.00 ± 9956.23	0.089
IGFBP3 pg/ml	643563.48 ±	616916.93 ± 24163.67	0.418
IGFBP4 ng/ml	404.91 ± 17.97	413.11 ± 11.22	0.711
IGFBP-7 pg/ml	15102.57 ±	16223.13 ± 419.22	0.181
IL10 pg/mL	1.32 ± 0.21	1.83 ± 0.22	0.097
Orexin-A pg/mL	77.44 ± 2.46	82.01 ± 3.25	0.254
TNF-α pg/mL	0.27 ± 0.08	0.39 ± 0.03	0.190
Leptin pg/ml	12406.96 ±	13110.23 ± 996.30	0.654
